# Quantum Hacking on an Integrated Continuous-Variable Quantum Key Distribution System via Power Analysis

**DOI:** 10.3390/e23020176

**Published:** 2021-01-30

**Authors:** Yi Zheng, Haobin Shi, Wei Pan, Quantao Wang, Jiahui Mao

**Affiliations:** School of Computer Science, Northwestern Polytechnical University, Xi’an 710129, China; shihaobin@nwpu.edu.cn (H.S.); panweihh@163.com (W.P.); wqt@nwpu.mail.edu.cn (Q.W.); maojiahui@nwpu.mail.edu.cn (J.M.)

**Keywords:** integrated continuous-variable quantum key distribution, quantum hacking, practical security

## Abstract

In quantum key distribution (QKD), there are some security loopholes opened by the gaps between the theoretical model and the practical system, and they may be exploited by eavesdroppers (Eve) to obtain secret key information without being detected. This is an effective quantum hacking strategy that seriously threatens the security of practical QKD systems. In this paper, we propose a new quantum hacking attack on an integrated silicon photonic continuous-variable quantum key distribution (CVQKD) system, which is known as a power analysis attack. This attack can be implemented by analyzing the power originating from the integrated electrical control circuit in state preparation with the help of machine learning, where the state preparation is assumed to be perfect in initial security proofs. Specifically, we describe a possible power model and show a complete attack based on a support vector regression (SVR) algorithm. The simulation results show that the secret key information decreases with the increase of the accuracy of the attack, especially in a situation with less excess noise. In particular, Eve does not have to intrude into the transmitter chip (Alice), and may perform a similar attack in practical chip-based discrete-variable quantum key distribution (DVQKD) systems. To resist this attack, the electrical control circuit should be improved to randomize the corresponding power. In addition, the power can be reduced by utilizing the dynamic voltage and frequency scaling (DVFS) technology.

## 1. Introduction

Quantum key distribution is an unconditionally secure quantum communication technology that promises that the authorized sender (Alice) and receiver (Bob) can share common keys through an insecure quantum channel in the presence of a potential eavesdropper (Eve) [1,2]. At present, discrete-variable quantum key distribution (DVQKD) and continuous-variable quantum key distribution (CVQKD) are two main categories of QKD systems that have been proved to be secure against general attacks (e.g., photon number splitting attacks on DVQKD and collective attacks on CVQKD) based on some basic assumptions [2,3,4,5]. Moreover, fiber-based QKD has been implemented by many research groups in laboratories and in field environments [6,7,8,9,10], and free-space QKD has been also studied experimentally. To further establish quantum communication networks, it is essential to explore high-performance and cost-effective QKD systems. Therefore, the implemented optical components of QKD systems were integrated on a silicon photonic chip by researchers in order to realize stable, miniaturized, and low-cost systems [11,12,13,14,15,16]. In particular, CVQKD with Gaussian-modulated coherent states (GMCS) is a widely studied protocol that has been integrated and realized [16]. Here, we focus on the exploration of chip-based GMCS CVQKD systems.

In the initial security proofs of QKD systems, the involved devices are modeled as secure and perfect. However, there are some imperfections in real-world QKD implementations that might open security loopholes for Eves to successfully steal secret key information [17,18]. These kinds of attacks are an effective quantum hacking strategy. For example, in practical DVQKD systems, an Eve may exploit some vulnerabilities in the single photon detector to launch a time-shift attack [19], an after-gate attack [20], a blinding attack [21], etc. Similarly, there are some quantum hacking attacks in practical CVQKD systems, such as the local oscillator (LO) fluctuation attack [22], LO calibration attack [23], wavelength attack [24,25], and saturation attack [26]. In addition, laser damage attacks and laser seeding attacks on the senders of QKD systems have been proposed [27,28,29,30]. It is important to note that these proposed quantum hacking attacks have corresponding countermeasures. The research on quantum attack and defense has effectively promoted commercial applications of QKD.

There is no doubt that chip-based QKD systems are also assumed to be perfect in security proofs. However, there are some new imperfections in practical chip-based QKD systems. For example, it is inevitable that the integrated electrical control circuit of a transmitter chip in a state preparation produces power associated with key information [31], which may open a new security loophole for Eves. In this work, we mainly investigate a possible quantum hacking attack exploited by this loophole in a chip-based GMCS CVQKD system. Based on the state preparation process in the transmitter of the system, we first modeled the power. The potential relation between the power and key information can be found by using some classical machine learning algorithms. Then, we exploited a support vector regression (SVR) algorithm to show the attack [32], which was composed of on-line and off-line stages. In the off-line stage, the same system was utilized by the Eve to collect power data in different periods. By using the SVR model to train these data, the aforementioned correlation could be obtained by the Eve, and could be exploited to analyze key information in a real-time chip-based GMCS CVQKD system. These analyses show a complete quantum hacking attack, which is named a power analysis attack. The simulation results indicate that the attack seriously destroys the practical security of the system. In particular, in low-noise environments, this impact is more obvious. Of course, a similar power analysis attack may be launched in practical chip-based DVQKD systems. Importantly, the power can be randomized by improving the electrical control circuit to effectively resist this attack. The dynamic voltage and frequency scaling (DVFS) technology can also be adopted to reduce the power to resist this attack. This study is of significance in promoting the establishment of quantum communication networks.

This paper is organized as follows: In Section 2, the power analysis attack is described and modeled. Then, we analyze the secret key rates of chip-based GMCS CVQKD systems under the effects of this attack in Section 3. To close the loophole opened by the power, some countermeasures are discussed in Section 4. Finally, conclusions are presented in Section 5.

## 2. Description of the Power Analysis Attack

**Figure 1:**
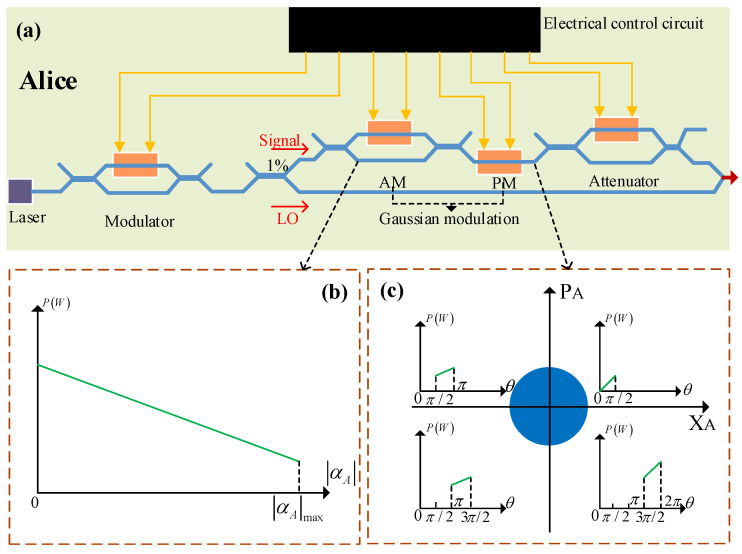
A possible power model of an integrated electrical control circuit in a Gaussian modulation of an integrated silicon photonic continuous-variable quantum key distribution (CVQKD) system, for which a similar relation in a classical chip has clearly been obtained [31]. Part (**a**) describes the transmitter of the chip-based CVQKD system. Part (**b**) shows the possible power in the amplitude modulation. Part (**c**) depicts the possible power in the phase modulation. AM, amplitude modulator; PM, phase modulator; LO, local oscillator.

Figure 1a shows the transmitter (Alice) of an integrated silicon photonic CVQKD system, where the involved optical components (except the laser source) are integrated on a silicon photonic chip [16]. In the chip, the first and last modulators serve attenuators to adjust the intensity of the optical signal. The other modulators (an amplitude modulator and a phase modulator) are exploited by Alice to generate a series of Gaussian-modulated coherent states $|\alpha_{A}\rangle_{u}\left(u=1,2,\ldots ,N\right)$ loaded with key information, where *N* is the total number of the generated states [7,16]. Based on the phase space, $|\alpha_{A}\rangle_{u}$ can be represented as

(1) $$\begin{array}{cc} \; & |\alpha_{A}\rangle_{u}={\left|\alpha_{A}\right|}_{u}e^{i\theta_{u}}=x_{A,u}+ip_{A,u},\\ \; & x_{A,u}=|\alpha_{A}{|}_{u}\cos\theta_{u},p_{A,u}={\left|\alpha_{A}\right|}_{u}\sin\theta_{u}, \end{array}$$

where $|\alpha_{A}\rangle_{u}$ and $\theta_{u}$ are the amplitudes and phases of these Gaussian-modulated states, respectively. In particular, $x_{A_{u}}$ and $p_{A_{u}}$ are random numbers that obey a Gaussian distribution $N(0,V_{A}N_{0}+N_{0})$. Here, $N_{0}$ is the variance of shot noise.

**Figure 2:**
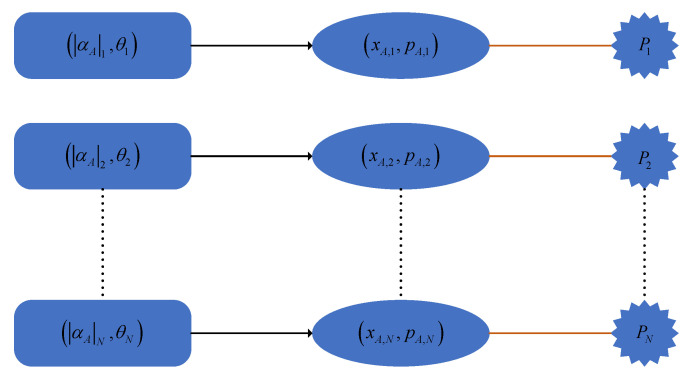
The correlation between the power and the key information in the state preparation.

In security proofs, the above state preparation is assumed to be perfect. However, it is inevitable that the integrated electrical control circuit of the transmitter chip generates power in the Gaussian modulation of practical chip-based CVQKD systems [31]. Here, the power produced by the integrated electrical circuit includes dynamic power $P_{dy}$ and static power $P_{st}$, where the dynamic power can be further divided into two parts: switching power $P_{sw}$ and short-circuit power $P_{sh}$. Moreover, $P_{sw}=C_{L}V_{dd}^{2}H_{t}f_{c}$, $P_{sh}=L{\left(V_{dd}-2V_{T}\right)}^{3}\tau f_{c}H_{t}$, $P_{st}=V_{dd}I_{leakage}$, where $C_{L}$ is the load capacitance, $V_{dd}$ is the supply voltage, $H_{t}$ is a trns factor, $f_{c}$ is the clock frequency, *L* is a technical parameter, $V_{T}$ is the threshold voltage, τ is the rise and fall time of the input signal, and $I_{leakage}$ is the leakage current [33]. In particular, the leakage current mainly includes the gate-induced drain leakage current, gate leakage current, reverse bias junction leakage current, and sub-threshold leakage current. These formulas for power indicate that the power is different when the operation statuses of the integrated control circuit are different. For the encoding of different key information, the required modulation voltages are different. Therefore, the power generated by the integrated electrical control circuit in the preparation process of these transmitted states should be different. Figure 1b depicts a possible power model during intensity modulation, where the power may decrease with the increase of the amplitude of the Gaussian-modulated coherent states. Figure 1c reveals a possible power model for phase modulation, where the power may be enhanced with the increase in the phase of the Gaussian-modulated states. According to Equation (1), the total power $P_{u}$ originating from the integrated electrical control circuit during Gaussian modulation should be hidden with a relation with random numbers $x_{A_{u}}$ or $p_{A_{u}}$, which is revealed in Figure 2. In particular, this relation is ambiguous in practical systems, and may be found by Eve through a classical machine learning algorithm. Therefore, the power originating from the integrated electrical control circuit of the transmitter of the practical chip-based CVQKD system may open a security loophole for Eve to successfully obtain key information, which seriously destroys the practical security of the system.

**Figure 3:**
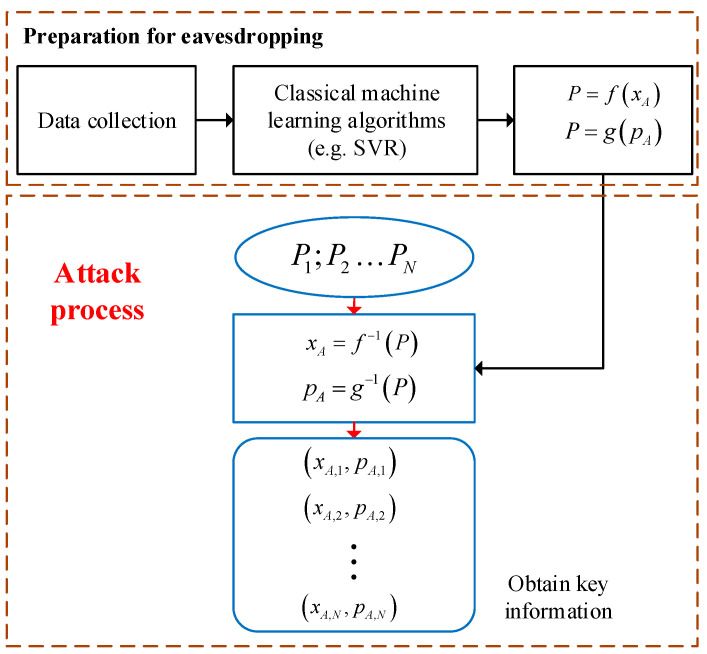
The process of the power analysis attack. SVR, support vector regression.

Figure 3 clearly introduces a complete power analysis attack, which includes two steps. The first step is off-line analysis. The purpose of this step is to explore the potential relationship between the key information and the power produced by the integrated electrical control circuit in state preparation. Specifically, Eve first utilizes an identical chip and a power meter to collect a series of power data originating from the integrated electrical control circuit in the preparation processes of different Gaussian-modulated coherent states, where Eve does not need to use some means to enter the transmitter chip. Then, some classical machine learning algorithms may be exploited by Eve to analyze the acquired data and get

(2) $$\begin{array}{cc} \; & P=f(x_{A}),\\ \; & P=g(p_{A}), \end{array}$$

where *P* is the power variable and $x_{A}$ and $p_{A}$ are two quadrature variables of the Gaussian-modulated optical signal. The above correlation may be nonlinear. Therefore, a support vector regression (SVR) algorithm may be exploited by Eve to analyze data, which can be modeled as [32]

(3) $$f\left(x_{A}\right)=W^{T}\Phi \left(x_{A}\right)+b,$$

where Φ(·) is a function that maps the input data into a higher dimensional space, W is the weight vector, and *b* is the bias. In order to achieve the optimal parameters W and *b*, the SVR model can be simplified as

(4) $$min\frac{1}{2}{\left\|W\right\|}^{2}+C\sum_{u=1}^{N}\left(\xi_{u}+\xi_{u}^{*}\right),C>0,$$

subject to

(5) $$\begin{array}{cc} \; & f\left(x_{A,u}\right)-P_{u}\leq \epsilon +\xi_{u},\\ \; & P_{u}-f\left(x_{A,u}\right)\leq \epsilon +\xi_{u}^{*},\\ \; & \xi_{u}\geq 0,\xi_{u}^{*}\geq 0,\epsilon >0, \end{array}$$

where *C* is a regularization parameter, and $\xi_{u}$ and $\xi_{u}^{*}$ respectively represent the upper and lower constraints in the outputs. In particular, ϵ is the permissible error. Then, W can be calculated as

(6) $$W=\sum_{u=1}^{N}\left(\lambda_{u}-\lambda_{u}^{*}\right)\Phi \left(x_{A,u}\right).$$

Here, $\lambda_{u}$ is the Lagrange multiplier. In addition, parameter *b* can also be calculated after W is obtained. According to Equations (3) and (6), the SVR model can be expressed by

(7) $$\begin{array}{cc} f(x_{A}) & =\sum_{u=1}^{N}\left(\lambda_{u}-\lambda_{u}^{*}\right)\Phi^{T}\left(x_{A,u}\right)\Phi \left(x_{A}\right)+b\\ \; & =\sum_{u=1}^{N}\left(\lambda_{u}-\lambda_{u}^{*}\right)k\left(x_{A,u},x_{A}\right)+b, \end{array}$$

where k(·,·) is a kernel function that includes three basic kernels: a polynomial kernel, linear kernel, and radial basis function (RBF). In general, the RBF kernel is a reasonable choice, as it is has low complexity and can solve the nonlinear relation. Here, the corresponding kernel function in Equation (7) should also be the RBF kernel, which is as follows:

(8) $$k\left(x_{A,u},x_{A}\right)=exp\{-\gamma |x_{A}-x_{A,u}{|}^{2}\}.$$

Here, γ indicates the scale parameter of the RBF kernel and determines model performance. In particular, the data collected by Eve in other time periods can serve as the test data. Based on the test data, the mean squared error (MSE) can be calculated as

(9) $$MSE=\frac{1}{n_{t}}\sum_{i=1}^{n_{t}}{\left[f\left(x_{t,i}\right)-P_{t,i}\right]}^{2},$$

where $n_{t}$ is the amount of the test data, and $x_{t,i}$ and $P_{t,i}$ are the values in test data. Here, MSE reflects the performance of the SVR algorithm. The smaller the value of the MSE, the better the performance of the algorithm. It is important to note that the potential relation between $p_{A}$ and the power *P* can also be explored by using the SVR model presented by Equations (3)–(8). When Equation (2) is acquired by Eve, she can further get

(10) $$\begin{array}{cc} \; & x_{A}=f^{-1}\left(P\right),\\ \; & p_{A}=g^{-1}\left(P\right). \end{array}$$

In a practical chip-based CVQKD system, Eve can exploit the acquired Equation (10) to steal key information by analyzing the power originating from the integrated electrical control circuit in state preparation, which is on-line analysis. This step is the core of the power analysis attack. Here, we define $P_{a}=1-MSE\left(0\leq MSE<1\right)$ as the accuracy of the power analysis attack that reflects the attack strength. In addition, when MSE≥1, the performance of the algorithm is poor, which indicates that the attack is ineffective. In particular, a similar attack can also be implemented in practical chip-based DVQKD systems.

## 3. Security Analysis

The performance of a chip-based CVQKD system can be measured by the secret key rate and the maximal transmission distance of the system. Given the parameters $V_{A}$, *T*, ε, η, and $\nu_{\mathrm{el}}$, the information shared by Alice and Bob can be calculated, as well as the maximal bound on the information available to the eavesdropper. Here, *T* and ε respectively represent the transmittance and excess noise of the quantum channel, which can be evaluated through parameter estimation. In addition, η and $\nu_{\mathrm{el}}$ are the detector’s fixed parameters, which respectively indicate the working efficiency and electronic noise. The secret key rate *K* with *n* received pulses used for key establishment against collective attacks is expressed as [18,22,27,34]

(11) $$K=\frac{n}{N}\left[\beta I_{AB}-S_{\mathrm{BE}}^{\epsilon_{\mathrm{PE}}}-\Delta \left(n\right)\right],$$

where reverse reconciliation and a finite-size effect are considered, n=N−m, *N* is the total number of the received pulses, *m* gives the values used for parameter estimation, β∈(0,1) is the reconciliation efficiency, $S_{\mathrm{BE}}^{\epsilon_{\mathrm{PE}}}$ represents the maximal value of the Holevo information compatible with the statistics except for probability $\epsilon_{\mathrm{PE}}$, and $I_{AB}$ represents the Shannon mutual information between Alice and Bob. Moreover, $\Delta \left(n\right)$ is a linear function of *n* that is related to the security of the privacy amplification. It can be given by [18,34]

(12) Δn=7log211ϵ¯ϵ¯n+2nlog21ϵPA,

where $\bar{\epsilon }$ and $\epsilon_{\mathrm{PA}}$, which are virtual parameters and can be optimized in the computation, denote the smoothing parameter and the failure probability of the privacy amplification, respectively. In addition, $\bar{\epsilon }$ and $\epsilon_{\mathrm{PA}}$ are usually set to be equal to $\epsilon_{\mathrm{PE}}$ because the value of Δ(n) mainly depends on *n*. It is important to note that the power analysis attack does not affect the transmitted states and the measurement of the received states. Therefore, the attack does not affect the parameter estimation, which indicates that Equations (11) and (12) cannot be destroyed by the attack.

According to the above analysis, the secret key rate of a system under a power analysis attack should be given by

(13) $$K_{P}=\left(1-P_{a}\right)\frac{n}{N}\left[\beta I_{AB}-S_{\mathrm{BE}}^{\epsilon_{\mathrm{PE}}}-\Delta \left(n\right)\right].$$

Here, $I_{AB}$ can be derived from Bob’s measured variance $V_{B}$ and the conditional variance $V_{B|A}$ as

(14) $$I_{AB}=\frac{1}{2}\log_{2}\frac{V_{B}}{V_{B|A}}=\frac{1}{2}\log_{2}\frac{V_{A}+1+\chi_{\mathrm{tot}}}{1+\chi_{\mathrm{tot}}},$$

where $\chi_{\mathrm{tot}}=\chi_{\mathrm{line}}+\chi_{\mathrm{hom}}/T$ represents the total noise referred to the channel input, $\chi_{\mathrm{line}}=1/T-1+\varepsilon$, and $\chi_{\mathrm{hom}}=\left[\left(1-\eta \right)+\nu_{\mathrm{el}}\right]/\eta$. In particular, $S_{\mathrm{BE}}^{\epsilon_{\mathrm{PE}}}$ is determined by the following covariance matrix between Alice and Bob with a finite-size effect:

(15) $$\begin{array}{cc} \; & \Gamma_{AB}=\\ \; & \left[\begin{array}{cc} \left(V_{A}+1\right)I & \sqrt{T_{\min}\left(V_{A}^{2}+2V_{A}\right)}\sigma_{z}\\ \sqrt{T_{\min}\left(V_{A}^{2}+2V_{A}\right)}\sigma_{z} & \left[T_{\min}\left(V_{A}+\varepsilon_{\max}\right)+1\right]I \end{array}\right], \end{array}$$

where matrices $I=\left[\begin{array}{cc} 1 & 0\\ 0 & 1 \end{array}\right]$ and $\sigma_{z}=\left[\begin{array}{cc} 1 & 0\\ 0 & -1 \end{array}\right]$. Here, $T_{\min}$ and $\varepsilon_{\max}$ respectively correspond to the lower bound of *T* and the upper bound of ε, which are defined as

(16) $$\begin{array}{cc} \; & T_{\min}={\left(t_{\min}\right)}^{2},\\ \; & \varepsilon_{\max}=\frac{\sigma_{\max}^{2}-1}{T}. \end{array}$$

According to Refs. [18,34], when *m* is large enough (e.g., $m>10^{6}$), $t_{\min}$ and $\sigma_{\max}^{2}$ can be calculated as

(17) $$\begin{array}{cc} \; & t_{\min}\approx \sqrt{T}-z_{\epsilon_{\mathrm{PE}}/2}\sqrt{\frac{1+T\varepsilon }{mV_{A}}},\\ \; & \sigma_{\max}^{2}\approx 1+T\varepsilon +z_{\epsilon_{\mathrm{PE}}/2}\frac{\left(1+T\varepsilon \right)\sqrt{2}}{\sqrt{m}}, \end{array}$$

where $z_{\epsilon_{\mathrm{PE}}/2}$ follows $1-\frac{1}{2}\mathrm{erf}\left(z_{\epsilon_{\mathrm{PE}}/2}/\sqrt{2}\right)=\frac{1}{2}\epsilon_{\mathrm{PE}}$, and erf(·) is the error function defined as $\mathrm{erf}\left(x\right)=\frac{2}{\sqrt{\pi }}\int_{0}^{x}e^{-t^{2}}dt$. Then, $S_{\mathrm{BE}}^{\epsilon_{\mathrm{PE}}}$ can be acquired by

(18) $$S_{\mathrm{BE}}^{\epsilon_{\mathrm{PE}}}=\sum_{i=1}^{2}G\left(\frac{\lambda_{i}-1}{2}\right)-\sum_{i=3}^{5}G\left(\frac{\lambda_{i}-1}{2}\right),$$

where $G\left(x\right)=\left(x+1\right)\log_{2}\left(x+1\right)-x\log_{2}x$, $\lambda_{i}\geq 1$ are symplectic eigenvalues derived from covariance matrices, which can be written as

(19) $$\begin{array}{cc} \lambda_{1,2}^{2}= & \frac{1}{2}\left(A\pm \sqrt{A^{2}-4B}\right),\\ \lambda_{3,4}^{2}= & \frac{1}{2}\left(C\pm \sqrt{C^{2}-4D}\right),\\ \lambda_{5}= & 1, \end{array}$$

where

(20) $$\begin{array}{cc} A & ={\left(V_{A}+1\right)}^{2}-2T_{\min}\left(V_{A}^{2}+2V_{A}\right)\\ \; & +{\left[T_{\min}\left(V_{A}+\varepsilon_{\max}\right)+1\right]}^{2},\\ B & ={\left[\left(T_{\min}\varepsilon_{\max}+1\right)\left(V_{A}+1\right)-T_{\min}V_{A}\right]}^{2},\\ C & =\frac{A\chi_{\mathrm{hom}}+\left(V_{A}+1\right)\sqrt{B}+T_{\min}\left(V_{A}+\varepsilon_{\max}\right)+1}{\eta T_{\min}\left(V_{A}+\varepsilon_{\max}\right)+1+\nu_{\mathrm{el}}},\\ D & =\frac{\sqrt{B}\left(V_{A}+1\right)+B\chi_{\mathrm{hom}}}{\eta T_{\min}\left(V_{A}+\varepsilon_{\max}\right)+1+\nu_{\mathrm{el}}}. \end{array}$$

Eventually, based on Equations (12)–(20), one can evaluate the secret key rate of a system under a power analysis attack.

**Figure 4:**
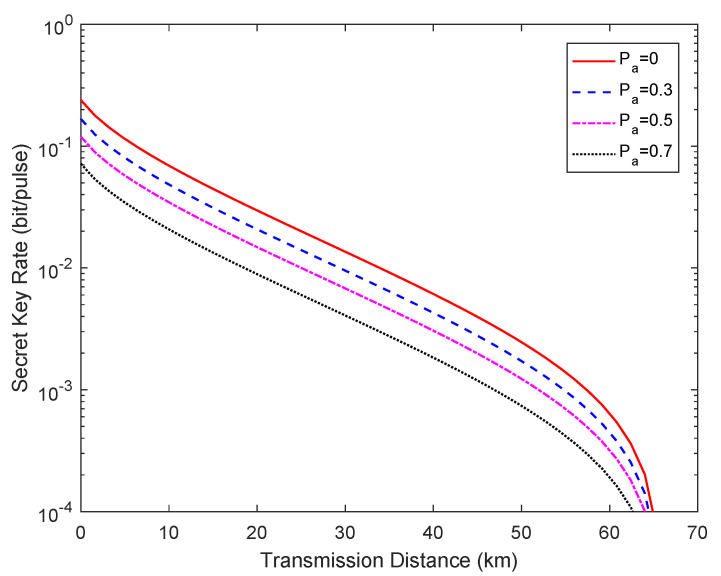
Secret key rate vs. transmission distance for different attack situations, where $P_{a}=0$ represents the ideal case without an attack. The fiber loss is 0.2 dB/km.

Figure 4 depicts the relationship between the secret key rate and the transmission distance for a practical chip-based CVQKD system under the effects of a power analysis attack when $P_{a}=0,0.3,0.5,0.7$. In particular, $P_{a}=0$ indicates that the attack was not carried out, i.e., the ideal case. The fixed parameters for the simulation are set as [16]: $V_{A}=7.07$ (in shot-noise units), η=0.498, $\nu_{\mathrm{el}}=0.0691$ (in shot-noise units), β=98%, ε=0.0934 (in shot-noise units), and $\epsilon =10^{-10}$, m=0.5×N, respectively. It is obvious that the secret key rate $K_{P}$ evaluated by Alice and Bob under the effects of the power analysis attack are reduced compared with the ideal value. The difference between the attacked secret key rate and the ideal value indicates the key information obtained by Eve.

**Figure 5:**
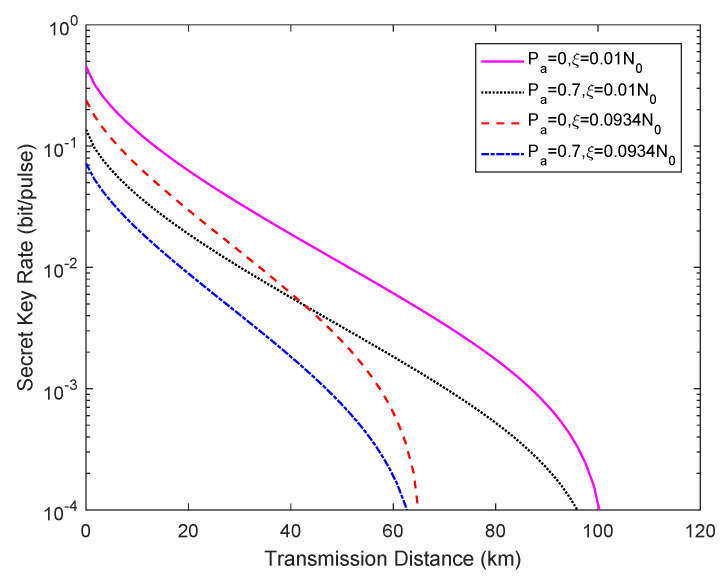
Secret key rate vs. transmission distance for different excess noise situations when $P_{a}=0.7$, where $P_{a}=0$ represents the ideal case without an attack.

Figure 5 describes the secret key rate versus the transmission distance under different excess noise levels (i.e., ε=0.0934,0.01) when $P_{a}=0.7$. The other simulation parameters remain unchanged. We find that Eve can acquire more secret key information in the case of less excess noise under the same attack strength.

More importantly, defending against power analysis attacks is a key task for establishing a quantum communication network, which is discussed in the next section.

## 4. Countermeasures

A complete power analysis attack is shown in the above analysis. The potential relation between key information and the power produced by the integrated electrical control circuit in state preparation is a security loophole exploited by Eve in the attack. Therefore, the electrical control circuit can be improved by randomizing the power to close this loophole, thus effectively resisting this attack. In addition, the pipeline structure and parallel structure can be adopted to optimize the electrical control circuit to reduce the power.

Apart from the above countermeasures, dynamic voltage and frequency scaling (DVFS) technology can be applied to reduce the dynamic power. The workflow of DVFS is as follows [35]:

Step 1: The signal related to system load is collected to calculate the current system load for the integrated electrical control circuit;

Step 2: Based on the current system load, the required performance is predicted for the control circuit system;

Step 3: The prediction performance is converted into the required frequency to adjust the clock setting of the integrated control circuit;

Step 4: According to the acquired frequency, the corresponding voltage can be obtained. Then, based on the acquired voltage, the central processing unit (CPU) voltage can be adjusted.

**Figure 6:**
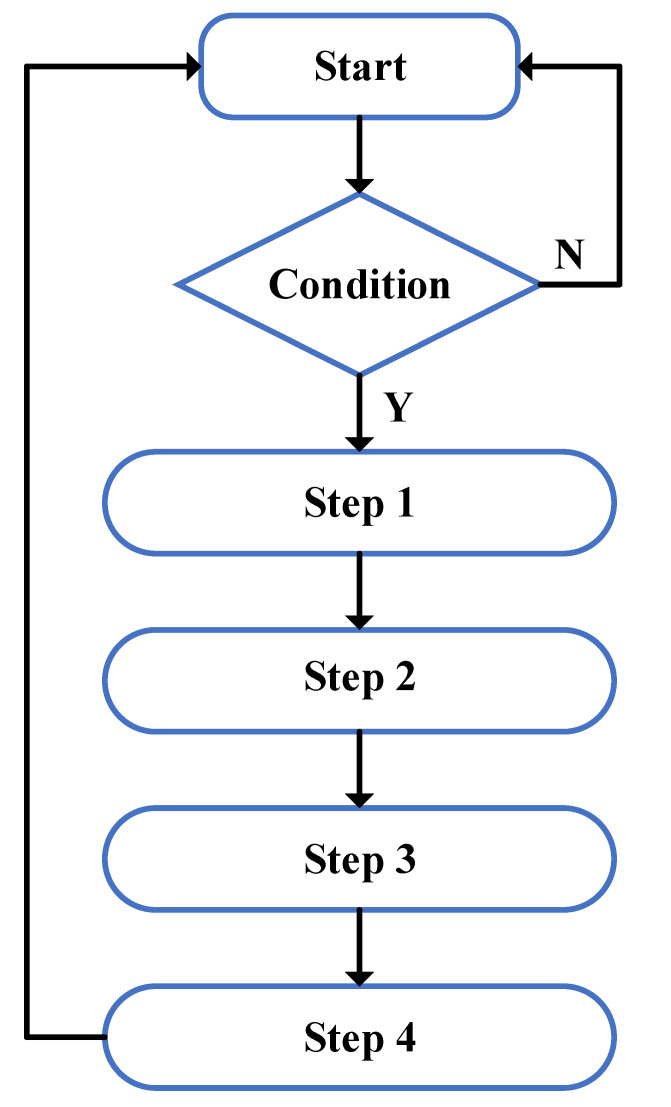
The flow of the dynamic voltage and frequency scaling (DVFS) algorithm. N, not; Y, yes.

Figure 6 shows a flowchart of a fast DVFS algorithm, where the judged condition is that the integrated control circuit sends data. As shown above, steps 1 to 4 have been described. In particular, the principle of the power produced by the integrated electrical control circuit in chip-based DVQKD systems is similar to that of integrated CVQKD systems. Therefore, these countermeasures can also be applied to resist similar attacks in chip-based DVQKD systems.

## 5. Conclusions

We have proposed a quantum hacking attack—namely, the power analysis attack—on an integrated silicon photonic CVQKD system. We first modeled the possible power originating from the integrated electrical control circuit in state preparation in the transmitter of the system, which clearly shows the correlation between the key information and the power. This correlation can be explored by Eve through some classical machine learning algorithms to steal key information, which indicates that the power produced by the electrical control circuit in state preparation can open a security loophole. Then, based on the SVR model, we showed a complete power analysis, which included off-line analysis and on-line real-time stealing. We found that Eve can acquire more key information in an environment with less excess noise through numerical analysis. In particular, a similar security loophole may also exist in chip-based DVQKD systems. Finally, electrical control circuits can be improved to effectively resist power analysis attacks. In addition, DVFS technology can also be applied to weaken the power. These countermeasures promote the application of QKD and the establishment of quantum communication networks.
